# THGC_MDA: a method for predicting the associations between m^1^A modification and diseases based on ternary heterogeneous network and graph convolutional neural network

**DOI:** 10.3389/fgene.2026.1806282

**Published:** 2026-03-20

**Authors:** Hongyan Gao, Xue Zhou, Lei Bai, Haifeng Yang, Fei Liu

**Affiliations:** School of Physics and Opto-Electronic Technology, Baoji University of Arts and Sciences, Baoji, Shaanxi, China

**Keywords:** graph convolutional neural network, m^1^A-disease associations, RNA modifications, self-attention mechanism, ternary heterogeneousnetwork

## Abstract

m^1^A modification, as a pivotal RNA epigenetic modification, plays a central regulatory role in the pathogenesis and progression of complex human diseases, including cancer. Exploring the potential associations between m^1^As and diseases are an important approach to revealing the molecular mechanism of disease onset. However, traditional biological experiments have the limitations of time-consuming and labor-intensive, resulting in an extremely scarce amount of verified m^1^A-disease association data. Meanwhile, the existing computational prediction methods are mostly limited to specific application scenarios and rely solely on the direct correlation data between m^1^As and diseases. They do not fully integrate multi-dimensional biological information and thus are unable to achieve efficient and accurate association predictions. In view of this, this study proposes a method for predicting the association between m^1^A modification and diseases based on a ternary heterogeneous network and GCN. By introducing circRNA as an intermediate connection node, a ternary association network of m^1^A-circRNA-disease is constructed, which effectively enriches the dimension of feature information for both m^1^A and diseases. Meanwhile, leveraging the feature learning capability of Graph Convolutional Network, the extraction and representation of their features are realized. The experimental results demonstrate that the proposed approaches significantly outperforms existing mainstream methods in predictive performance, substantially enhancing the accuracy and reliability of m^1^A-disease association prediction. Furthermore, case validation has further confirmed that the predicted candidate m^1^A sites participate in regulating disease-related gene expression networks by modulating core processes such as RNA localization, stability, and translation efficiency, thereby providing novel insights into the investigation of disease pathogenesis.

## 1 Introduction

N1-methyladenine (m^1^A) is a reversible post-transcriptional RNA modification widely present in tRNA, rRNA and mRNA of eukaryotes. By regulating RNA stability, translational capacity, and protein function, m^1^A exerts a regulatory effect on disease progression (Zhang and Jia, 2018). The research team from the School of Pharmaceutical Sciences, Sun Yat-sen University, demonstrated that m^1^A modulates the expression of ATP5D (the δ subunit of mitochondrial ATP synthase), thereby controlling the glycolytic activity of tumor cells (Wu et al., 2022). This discovery not only reveals the critical role of m^1^A modification in cancer metabolism but also underscores its broader implications in the pathogenesis of human diseases, particularly cancer.

In recent years, an increasing number of studies have been dedicated to elucidating the biological functions and regulatory mechanisms of m^1^A modification in the initiation and progression of diseases. Consequently, many computational tools facilitating the prediction of m^1^A functions have been developed. Dominissini et al. (2016) for the first time mapped the dynamic m^1^A methylation patterns in eukaryotic mRNA through the integration of chemical enrichment and high-throughput sequencing. Wang et al. (2021) employed LC-MS/MS quantification, CRISPR-mediated knockout of TRMT6/61A, RNA-seq and cholesterol tracing to verify that m^1^A modification promotes hepatocellular carcinoma (HCC) initiation by regulating cholesterol metabolism.

Currently, researchers have compiled databases of RNA chemical modification information, such as RNAMDB (Cantara et al., 2011), RMBASE (Xuan et al., 2018), MeT-DB (Liu et al., 2015), and m^6^AVAR (Zheng et al., 2018), which have facilitated subsequent research on RNA chemical modifications. The method for predicting the associations between RNA modifications and diseases constitutes a complex and rapidly evolving field. Notably, the exploration of unknown correlations based on the known associations derived from existing datasets has received significant attention. Graph neural networks (GNN) have emerged as powerful deep learning architectures for modeling graph-structured data in biological systems. By iteratively aggregating feature information from neighboring nodes through message passing mechanisms, GNN can effectively learn node embeddings that capture both local graph topology and node attributes. More recently, a comprehensive review by Khemani et al. (Khemani et al., 2024) further summarized the fundamental concepts, architectures, and training techniques of GNNs, and discussed their challenges, benchmark datasets, and diverse applications, highlighting the rapid development and broad applicability of GNN-based approaches across multiple domains. Ma et al. (2021a) integrated the positional information of m^7^G and comprehensive disease similarity information to construct a heterogeneous network. Then, the matrix decomposition method was applied to predict potential disease-related m^7^G sites. Huang et al. (2024) applied artificial intelligence–based approaches to investigate epitranscriptome distribution, providing new insights into the landscape of RNA modifications. Zhang Y. et al. (2025) developed DirectRM, a framework that enables the integrated detection of multiple RNA modifications and their potential crosstalk using direct RNA sequencing. Huang et al. (2023) proposed a computational framework based on random walk in heterogeneous networks. By integrating multi-dimensional information such as RNA sequences, structural features, expression profiles, and disease phenotypes, to construct a ternary m^7^G-RNA-disease network. Through the random walk algorithm, the associated signals are globally propagated to predict potential disease-related m^7^G modification sites. Liu et al. (2023) proposed RMDGCN, a graph convolutional network integrated with an attention mechanism, to predict the relationship between m^1^A modifications and diseases. Zhang and Liu (2025) proposed m6ADP-GCNPUAS, a method that predicts the association between m^6^A and diseases through graph convolutional neural networks (GCNs) and Positive-Unlabeled Learning with Self-Adaptive Sampling (PUAS). In addition, several methods developed for predicting the associations between circRNAs and diseases can provide valuable insights for our research. Wang et al. (2020a) employed deep convolutional neural networks to explore the associations between circRNA and diseases. The framework of their method, including feature extraction and application of machine learning models, provided insights for the prediction of RNA modifications and disease associations. Wang et al. (2020b) proposed GCNCDA based on graph convolutional networks. By integrating Gaussian interaction profile kernels and similarity networks of both circRNAs and diseases, they constructed a two-layer graph convolutional network to automatically learn node embeddings, then followed by inner product decoding to predict potential circRNA- diseases associations. Bian et al. (2021) introduced GATCDA, which constructed a graph attention network to adaptively learn neighbor weights and aggregated node features, and then predicted circRNA-disease associations through a bilinear decoder. Despite the availability of these prediction methods, most of them solely rely on RNA modification-disease association data and fail to integrate additional datasets to explore the underlying mechanisms of the relationship between RNA modifications and diseases. Therefore, it is necessary to develop a novel computational tool to extract more complex m^1^A features related to diseases and apply them to a broader range of scenarios.

Based on this, this paper proposes a method for predicting m^1^A modification-disease associations using a ternary heterogeneous network and graph convolutional network, termed THGC_MDA. This model introduces circRNA as an intermediary to construct a ternary heterogeneous network of m^1^A-circRNA-disease. It extracts the features of m^1^A and diseases respectively through the graph convolutional network, thereby realizing the prediction of their associations.

## 2 Materials and methods

**FIGURE 1:**
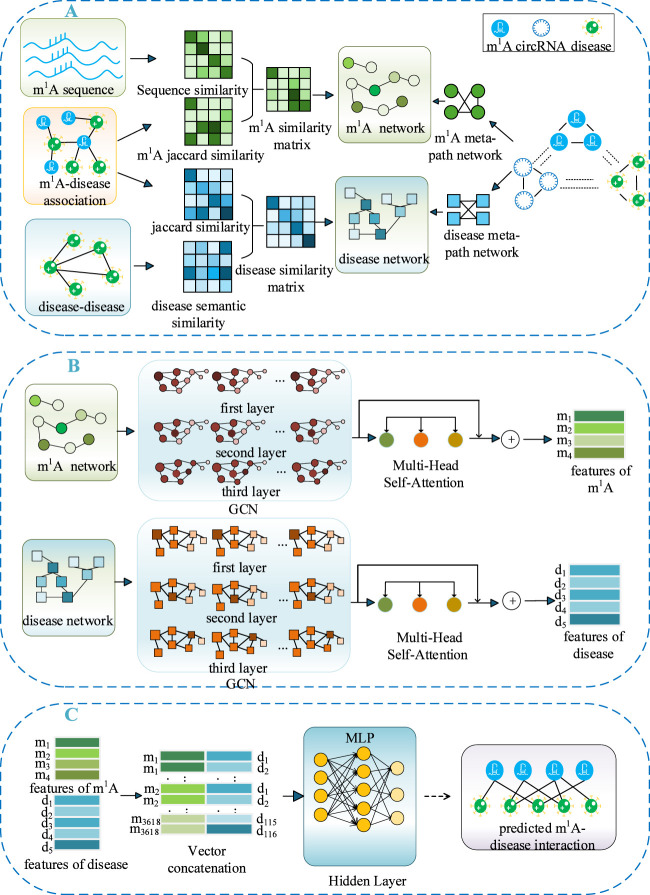
The flowchart of THGC_MDA. **(A)** Construction of the m^1^A-disease heterogeneous graph and meta-paths for feature extraction. **(B)** Deep feature learning via multi-layer GCN. **(C)** Prediction of potential m^1^A-disease associations using MLP.

Figure 1 illustrates the specific workflow of THGC_MDA. Initially, this model constructs an m^1^A similarity network based on the m^1^A sequence and Jaccard similarity. Subsequently, it establishes a similarity matrix for diseases by integrating the Jaccard similarity and semantic similarity of the diseases. Concurrently, by leveraging the ternary heterogeneous network of m^1^A-circRNA-disease, the meta-path networks of m^1^A and diseases were respectively constructed. Then, through a multi-layer GCN, deep feature learning is performed on both m^1^A and diseases to obtain their respective updated feature representations. Finally, the features of both are combined to output a prediction score matrix for m^1^A -disease associations. As illustrated in Figure 1, panel (A) shows the construction of the m^1^A-disease heterogeneous graph, in which we separately integrate m^1^A and disease similarities and employ circRNA to establish meta-paths for extracting the features of m^1^A sites and diseases. Panel (B) describes the deep feature learning process implemented via multi-layer GCN. Panel (C) presents the prediction of potential associations between m^1^A and diseases using a MLP.

### 2.1 Dataset

The data on the association between m^1^A and diseases used in this study is the same as that in RMDGCN(14), both sourced from the RMVar database (http://rmvar.renlab.org) (Luo et al., 2021). To ensure data credibility, we retained modified sites with high confidence and disease information with corresponding Disease Ontology Identifiers (DOID) from the Disease Ontology database. Finally, 3,618 pieces of mutation-related m^1^A modification sites, 116 diseases, and 5,100 associations were obtained. Furthermore, in order to construct the ternary heterogeneous network of m^1^A-circRNA-diseases, we mapped 3,618 m^1^A modification sites onto circRNAs, resulting in a total of 222 circRNAs. Additionally, we obtained 1,022 relationships between 222 circRNAs and 116 diseases.

### 2.2 Similarity calculation

This paper applies semantic similarity and Jaccard similarity to diseases, and applies sequence similarity and Jaccard similarity to m^1^A.

#### 2.2.1 Construction of the disease similarity network

We retrieved the Disease Ontology identifiers (DOID) corresponding to each disease from the Disease Ontology dataset (Kibbe et al., 2015), which were utilized for calculating the semantic similarity of diseases. The similarity was computed using the Dosim function provided in the R language package (Yu et al., 2014). In the disease ontology framework, each disease is represented by a directed acyclic graph (DAG). The disease semantic similarity between disease *i* and disease *j* can be defined as follows in Equation 1:

$$SSD\left(d_{i},d_{j}\right)=\frac{\sum_{f\in Fc_{i}\cap Fc_{j}}\left(Dc_{i}\left(t\right)+Dc_{j}\left(t\right)\right)}{\sum_{f\in Fc_{i}}Dc_{i}\left(t\right)+\sum_{f\in Fc_{j}}Dc_{j}\left(t\right)}$$ (1)

Among them, $F_{c_{i}}$ and $F_{c_{j}}$ are the ancestor nodes of disease *i* and disease *j* respectively, $D_{c_{i}}\left(t\right)$ represents the contribution value of all nodes in $F_{c_{i}}$ to disease *i*, and is defined as Equation 2:

$$Dc_{i}\left(t\right)=\left\{\begin{array}{l} 1,\\ \max\{\alpha \cdot Dc_{i}\left(t^{\prime }\right)\left|t^{\prime }\in children\left(t\right),\begin{array}{cc} \;if & t \end{array}\neq d\},\right. \end{array}\right.$$ (2)

Among them, $children\left(t\right)$ represents the child nodes belonging to *t*; *α* is the disease semantic score, and the default setting is 0.5 (Rashid et al., 2025).

In the Disease Ontology framework, diseases that share similar ancestor nodes in the DAG structure tend to have related pathological mechanisms or biological characteristics. Therefore, diseases with higher semantic similarity scores are more likely to share similar molecular mechanisms or regulatory processes. Incorporating disease semantic similarity into the model helps capture functional relationships between diseases and provides biologically meaningful information for identifying potential associations between m^1^A modifications and diseases.

Given the sparsity of disease semantic similarity data, which limits the comprehensive representation of disease features, the Jaccard similarity is used to enrich the similarity information.

The Jaccard similarity between diseases *d*_*i*_ and *d*_*j*_ is calculated based on the corresponding columns in the association matrix as follows in Equation 3:

$$JJD\left(d_{i},d_{j}\right)=\frac{y_{i}\cdot y_{j}}{{\left\|y_{i}\right\|}_{1}+{\left\|y_{j}\right\|}_{1}-y_{i}\cdot y_{j}}$$ (3)

Among them, *y*_*i*_ and *y*_*j*_ correspond to the feature vectors (row vectors or column vectors) of disease *i* and disease *j* in the association matrix (they must be consistent), and the elements of the vectors are usually 0 or 1; *||y*_*i*_ *||*_1_ and *||y*_*j*_ *||*_1_ represent the L1 norm of vectors *y*_*i*_ and *y*_*j*_ (that is the sum of all elements in the vectors), and they are respectively equal to the total number of features of disease *i* and disease *j*.

From a biological perspective, diseases that share more common associations in the interaction network are more likely to exhibit similar pathological mechanisms or molecular regulatory processes. Therefore, the Jaccard similarity reflects the degree of overlap between the association patterns of two diseases. A higher Jaccard similarity score indicates that the two diseases share more common interaction partners, suggesting potential functional or mechanistic relationships. Incorporating Jaccard similarity helps capture structural relationships within the disease network and complements the disease semantic similarity in representing disease-related features.

#### 2.2.2 Construction of the m^1^A similarity network

To calculate the sequence similarity between the m^1^A modification sites, we first extracted 65-nt RNA sequence fragments from the reference sequence, with each fragment containing an m^1^A site at the center and flanked by 32 nucleotides upstream and 32 nucleotides downstream. In computational studies of RNA modification sites, extracting fixed-length sequence windows centered on the modification site is a widely used method to capture the local sequence environment surrounding the modified nucleotide. Previous studies have demonstrated that the nucleotide sequences flanking RNA modification sites contain important sequence features that are informative for identifying RNA modification sites (Chen et al., 2020). For example, Zhou et al. (2016) developed the predictor SRAMP, which employed a 61-nt sequence window to characterize the sequence context surrounding m^6^A modification sites. Following these studies, we adopted a window size of 65 nt (32 nt upstream and 32 nt downstream of the central site), which is sufficient to capture the local contextual sequence information around the modification site while maintaining a consistent input length for downstream computational analysis. Subsequently, these sequence fragments were subjected to one-hot encoding, which each base (A, U, G, C) was mapped to a four-dimensional binary vector (for example, A was represented as [1,0,0,0], U as [0,1,0,0], G as [0,0,1,0], and C as [0,0,0,1]) (Wang et al., 2024). Given that each sequence is 65 nt in length, each sequence was represented as a high-dimensional vector with a dimension of 65 × 4 = 260 following encoding.

After performing one-hot encoding, we adopted cosine similarity as the metric to calculate the similarity among all sequences corresponding to the m^1^A sites. The cosine similarity value between two m^1^A sites is defined as Equation 4:

$$SSM\left(i,j\right)=\frac{m_{i\cdot }m_{j}}{{\left\|m_{i}\right\|}_{2}\times {\left\|m_{j}\right\|}_{2}}=\frac{\sum_{k=1}^{d}m_{i,k}\times m_{j,k}}{\sqrt{\sum_{k=1}^{d}{\left(m_{i,k}\right)}^{2}}\times \sqrt{\sum_{k=1}^{d}{\left(m_{j,k}\right)}^{2}}}$$ (4)

Among them, *SSM(i,j)* denotes the cosine similarity between m^1^A site *i* and m^1^A site *j*. Specifically, ***m***_*i*_ and ***m***_*j*_ represent the sequence embedding feature vectors of m^1^A site *i* and m^1^A site *j* respectively. Here, *d* represents the total dimension of the vector derived from one-hot encoding of each sequence. Meanwhile, $m_{i,m}$ and $m_{j,m}$ represent the *m*-th element of the one-hot encoding vectors corresponding to sequence of m^1^A site *i* and m^1^A site *j*, respectively. Then, the numerator represents the dot product of the two vectors, calculated by multiplying each pair of corresponding elements and summing the results. The denominator is the product of the L2 norms of the two vectors, where the L2 norm of a vector is defined as the square root of the sum of the squares of its individual elements.

From a biological perspective, sequence similarity reflects the degree of conservation in the nucleotide context surrounding m^1^A modification sites. m^1^A modifications often occur within specific sequence environments or motifs. Therefore, m^1^A sites with similar sequence patterns in their flanking regions may share similar structural characteristics or regulatory mechanisms involved in RNA modification. By measuring sequence similarity, the model can capture the local sequence features associated with m^1^A modification sites, which provides biologically meaningful information for identifying potential relationships among modification sites.

Furthermore, the Jaccard similarity is also employed to enrich the similarity information of m^1^A modification sites. The Jaccard similarity between vectors *m*_*i*_ and *m*_*j*_ (corresponding to m^1^A sites *i* and *j*) is calculated based on the corresponding rows in the association matrix, as show below Equation 5:

$$JJM\left(m_{i},m_{j}\right)=\frac{x_{i}\cdot x_{j}}{{\left\|x_{i}\right\|}_{1}+{\left\|x_{j}\right\|}_{1}-x_{i}\cdot x_{j}}$$ (5)

Among them, ***x*** *ᵢ* and ***x***_*j*_ correspond to the eigenvectors (row vectors or column vectors, with consistency required) of the m^1^A features in the association matrix corresponding to the *i*-th and *j*-th m^1^A respectively. The elements of these vectors are usually typically binary(0 or 1). *||* ***x*** *ᵢ||*_1_ and *||* ***x***_*j*_ *|*|_1_ denote the L1-norms of vectors ***x*** *ᵢ* and ***x***_*j*_ (i.e., the sum of all elements in each vector), which are equivalent to the total number of features associated with the *i*-th m^1^A and the *j*-th m^1^A sites.

#### 2.2.3 Fusion of similarity networks

In order to better integrate the similarities of diseases, *DDS* represents the ultimate similarity of diseases, and the formula is as follows in Equation 6:

$$DDS=\frac{SSD+JJD}{2}$$ (6)

Among them, *SSD* denotes the semantic similarity of diseases, and *JJD* represents the Jaccard similarity of diseases. The final disease similarity is derived by averaging these two similarity metrics. Similarly, for m^1^A similarity in Equation 7, *SSM* is the sequence similarity of m^1^A, *JJM* denotes the Jaccard similarity, and the average of these similarities is used to obtain the final similarity of m^1^A.

$$DDM=\frac{SSM+JJM}{2}$$ (7)

#### 2.2.4 Construction of the meta-path network

The meta-path can comprehensively connect m^1^A, circRNA and diseases together, while capturing detailed structural information within the correlation network (Zhang X. et al., 2025). Based on the meta-path framework, we constructed two distinct adjacency matrices to capture network structural information at multiple hierarchical levels. The meta-path network of m^1^A is as show in Equation 8:

$$A_{m1}=A_{MC}\times A_{MC}^{T}$$ (8)

$$A_{m2}=A_{MC}\times A_{CD}\times A_{CD}^{T}\times A_{MC}^{T}$$ (9)

Among them, $A_{MC}$ denotes the m^1^A-circRNA association matrix, $A_{CD}$ represents the circRNA-disease association matrix, and $A_{m1}$ quantifies association strengths between m^1^A nodes through shared circRNA neighbors, while $A_{m2}$ captures indirect associations via the m^1^A-circRNA-disease pathway in Equation 9. $A_{MC}^{T}$ represents the transposed association network of m^1^A and circRNA, and $A_{CD}^{T}$ represents the transposed association network of circRNA and diseases.

Similarly, the network representing the root causes of the disease is as show in Equation 10:

$$A_{d1}=A_{CD}^{T}\times A_{CD}$$ (10)

$$A_{d2}=A_{CD}^{T}\times A_{MC}^{T}\times A_{MC}\times A_{CD}$$ (11)

where, $A_{d1}$ measures association strengths between disease nodes through shared circRNA neighbors, and $A_{d2}$ encodes indirect associations via the disease-circRNA-m^1^A pathway in Equation 11.

### 2.3 Graph convolutional network

We further utilized the graph convolutional network (GCN) to extract the embedding features of each m^1^A and disease nodes. GCN fuses the feature vectors of neighboring nodes with the input m^1^A-m^1^A or disease-disease network (encoded as an adjacency matrix), effectively learning the embedding features of each node (Wu et al., 2020). The GCN architecture requires two core inputs: an initial node feature matrix and an adjacency matrix defining the graph structural topology. For m^1^A nodes, the initial feature matrix consists of enhanced representations generated via similarity fusion and feature enrichment. For disease nodes, the feature matrix integrates semantic and Jaccard similarity measures. The adjacency matrix encodes the connectivity patterns between nodes in the network.

For m^1^A nodes, the adjacency matrix integrates information from both the m^1^A-circRNA association path $A_{m1}$ , the m^1^A -circRNA-disease association path $A_{m2}$ , and the m^1^A similarity matrix *DDM*, formulated as in Equation 12:

$$M_{1}=0.6\times A_{m1}+0.3\times A_{m2}+0.1\times DDM$$ (12)

The weighting coefficients (0.6, 0.3, 0.1) prioritize direct neighbor relationships while incorporating higher-order connectivity information. The coefficient 0.6 corresponds to first-order neighborhood aggregation (direct connectivity via shared circRNA), preserving local structural information analogous to standard GCN designs. The coefficient 0.3 corresponds second-order cross-modal propagation (bridging m1A and disease through two-hop circRNA paths). The coefficient 0.1 indicates that the similarity matrix serves as feature preprocessing and prior regularization.

For disease nodes, the adjacency matrix is constructed by integrating the circRNA-disease association path $A_{d1}$ , the disease-circRNA- m^1^A association path $A_{d2}$ , and the disease similarity matrix *DDS* in Equation 13:

$$M_{2}=0.6\times A_{d1}+0.3\times A_{d2}+0.1\times DDS$$ (13)

The constructed adjacency matrices undergo symmetric normalization to ensure numerical stability during feature propagation in Equations 14, 15:

$${\tilde{M}}_{1}=D_{1}^{-\frac{1}{2}}M_{1}D_{1}^{-\frac{1}{2}}$$ (14)

$${\tilde{M}}_{2}=D_{2}^{-\frac{1}{2}}M_{2}D_{2}^{-\frac{1}{2}}$$ (15)

where $D_{1}$ and $D_{2}$ represent the degree matrices of $M_{1}$ and $M_{2}$ , respectively. These diagonal matrices contain elements $D_{1,ii}=\sum_{j}M_{1,ij}$ and $D_{2,ii}=\sum_{j}M_{2,ij}$ , and the normalization process mitigates issues of gradient explosion or vanishing during network training.

The multi-layer GCN architecture learns hierarchical embedding representations $H^{\left(l\right)}$ for both m^1^A nodes and disease nodes. For m^1^A nodes, the feature transformation at the l -th layer is defined as in Equations 16, 17:

$$H_{m^{1}A}^{\left(l\right)}=\text{ReLU}\left({\tilde{M}}_{1}H_{m^{1}A}^{\left(l-1\right)}W_{m^{1}A}^{\left(l-1\right)}\right)$$ (16)

$$\text{ReLU}\left(x\right)=\max\left(0,x\right)$$ (17)

where $H_{m^{1}A}^{\left(l-1\right)}$ denotes the m^1^A node features at the l−1 -th layer, $W_{m^{1}A}^{\left(l-1\right)}$ represents the trainable weight matrix, and ReLU is the Rectified Linear Unit activation function introducing non-linearity.

Similarly, for disease nodes, the feature update follows in Equation 18:

$$H_{\text{dis}}^{\left(l\right)}=\text{ReLU}\left({\tilde{M}}_{2}H_{\text{dis}}^{\left(l-1\right)}W_{\text{dis}}^{\left(l-1\right)}\right)$$ (18)

where $H_{\text{dis}}^{\left(l-1\right)}$ contains the disease node features at the l−1 -th layer and $W_{\text{dis}}^{\left(l-1\right)}$ is the corresponding trainable weight matrix.

To enhance the model’s representational capacity, we incorporated a multi-head self-attention mechanism with residual connections following the GCN feature learning. The self-attention mechanism adaptively computes importance weights between nodes in Equation 19:

$$\text{Attention}\left(Q,K,V\right)=\text{softmax}\left(\frac{QK^{T}}{\sqrt{d_{k}}}\right)V$$ (19)

$$\text{softmax}\left(z_{i}\right)=\frac{e^{z_{i}}}{\sum_{j}e^{z_{j}}}$$ (20)

where Q , K and V denote the query, key, and value matrices obtained through linear transformations of node features, $d_{k}$ represents the key vector dimension, and the softmax function computes normalized attention weights in Equation 20.

Residual connections facilitate gradient flow by combining attention outputs with original GCN features in Equation 21:

$$H_{final}=H_{attention}+W_{residual}H_{GCN}$$ (21)

where $W_{residual}$ is a learnable residual weight matrix.

For association prediction, we implemented a feature interaction network that processes the final embeddings of m^1^A and disease nodes. The m^1^A feature $h_{i}$ and disease feature $h_{j}$ are concatenated and processed through a multi-layer perceptron in Equation 22:

$$h_{\text{interaction}}=\text{MLP}\left(\left[h_{i}\|h_{j}\right]\right)$$ (22)

where ‖ denotes vector concatenation and MLP comprises multiple non-linear transformation layers.

The interaction features are then projected through a prediction network to generate association probabilities in Equations 23, 24:

$$p_{ij}=\sigma \left(W_{p}h_{\mathrm{int}eraction}+b_{p}\right)$$ (23)

$$\sigma \left(x\right)=\frac{1}{1+e^{x}}$$ (24)

where σ represents the Sigmoid activation function, $W_{p}$ and $b_{p}$ are the weight matrix and bias term, respectively, and the output is constrained to the [0,1] interval, representing the probability of association between m^1^A and the disease.

To address class imbalance, we employed a composite loss function in Equations 25-27:

$$L=0.7\times L_{BCE}+0.3\times L_{Focal}$$ (25)

$$L_{BCE}=-\frac{1}{N}\sum_{i=1}^{N}\left[y_{i}\log\left(p_{i}\right)+\left(1-y_{i}\right)\log\left(1-p_{i}\right)\right]$$ (26)

$$L_{Focal}=-\frac{1}{N}\sum_{i=1}^{N}\alpha {\left(1-p_{i}\right)}^{\gamma }y_{i}\log\left(p_{i}\right)+\left(1-\alpha \right)p_{i}^{\gamma }\left(1-y_{i}\right)\log\left(1-p_{i}\right)$$ (27)

where $L_{BCE}$ denotes binary cross-entropy loss, $L_{Focal}$ represents Focal Loss with parameters α=0.25 and γ=2 , which dynamically adjusts sample weights to prioritize challenging examples during training.

This comprehensive GCN architecture enables effective learning of deep feature representations for m^1^A and diseases within the heterogeneous network, facilitating accurate prediction of m^1^A-disease associations.

## 3 Results and discussion

### 3.1 Evaluation metrixs

The model was evaluated via 5-fold cross-validation. In the 5-fold cross-validation process, the complete set of m^1^A-disease associations was split into five equal sized parts. In each cross-validation, one part was used as the test set, and the remaining four parts were used as the training set. In order to better evaluate the performance of the model, we use *ACC*, *Precision*, *Recall*, *F1_score*, *AUPR* and *AUC* to present the results of the model. Their definitions are as follows in Equation 28:

$$\begin{array}{l} TPR=\frac{TP}{TP+FN}\\ FPR=\frac{TN}{N}=\frac{TN}{TN+FP}\\ ACC=\frac{TP+TN}{TP+TN+FP+FN}\\ Recall=\frac{TP}{TP+FN}\\ Precision=\frac{TP}{TP+FP}\\ F1\_score=\frac{2\times Precision\times Recall}{Precision+Recall} \end{array}$$ (28)

Among them, *TP* stands for true positive, *TN* for true negative, *FP* for false positive, and *FN* for false negative. For the Receiver Operating Characteristic (ROC) curve, the vertical axis is the true positive rate (*TPR*), while the horizontal axis is the false positive rate (*FPR*). *AUC* is the area under this curve with a value ranging from 0 to 1, the closer it is to 1, the stronger the model’s ability to distinguish between positive and negative classes, thus achieving more accurate predictions. The Precision-Recall (PR) curve takes *Recall* as its horizontal axis and *Precision* as its vertical axis. The Area Under the PR Curve (*AUPR*) corresponds to the area under this curve. It can better reflect the true performance of the model when there are few positive samples.

### 3.2 Comparing with other methods

This section compares THGC_MDA with six other methods to evaluate the performance advantage in predicting m^1^A-diseases associations. The comparative results are primarily derived from RMDGCN (Liu et al., 2023), a computational method based on the attention mechanism and GCN to predict the associations between RNA methylation and diseases. The raw data utilized in this paper is the same as that in RMDGCN. Furthermore, to verify the superiority of THGC_MDA, both the comparative method and evaluation metrics employed herein are consistent with those adopted in RMDGCN.

RWR (Tong et al., 2006) can utilize node information through multiple approaches, capturing global information of the graph to obtain the correlation scores between nodes. Given that a single molecule can induce various diseases, the potential factors governing the molecular-disease associations are highly correlated. NMF (Lee and Seung, 1999) decomposes the correlation matrix into a basis matrix and a weight matrix. The basis matrix represents the relative contribution of potential factors, while the weight matrix represents the relative contribution of the diseases. Additionally, we compared three existing methods: BRPCA (Ma et al., 2021b) for m^7^G-disease associations prediction, SCMDDF (Zhang et al., 2018) for drug-disease associations prediction, and LOMDA (Pech et al., 2019) for miRNA-disease associations prediction. BPRCA employs singular value decomposition to impute missing items in the adjacency matrix of the heterogeneous network, thereby obtaining the fitting matrix for the repaired associations between modification sites and diseases. SCMDDF predicts drug-disease associations through similarity constraint matrix decomposition, while LOMDA is a linear prediction method for miRNA-disease relationships based on linear optimization.

**FIGURE 2:**
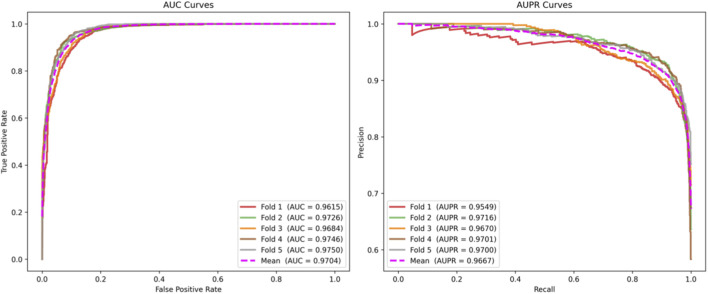
The ROC curves and PR curves for 5CV.

The 5-fold cross-validation results demonstrate that THGC_MDA outperforms other competing methods. Detailed performance metrics for each evaluation index are summarized in Table 1. Figure 2 presents the 5-fold cross-validation results of AUC and AUPR for THGC_MDA, along with the corresponding ROC curves and PR curves derived from the final model. To address class imbalance, we implemented a random negative sampling strategy: for all experimentally validated m^1^A-disease associations (positive samples), an equal number of unconnected m^1^A-disease pairs were randomly selected from the complete bipartite space as negative samples, yielding a balanced 1:1 training dataset. The GCN model was configured with the following hyperparameters: 300 training epochs, initial learning rate of 0.001, GCN encoder hidden dimension of 256, embedding output dimension of 128, and a 3-layer multilayer perceptron (MLP) predictor (32→64→32→1) with Sigmoid activation for probability calibration.

**TABLE 1:** Performance comparison of different methods.

Methods	AUC	AUPR	ACC	Recall	Precision	F1_score
RWR	0.6343	0.0694	0.9036	0.1900	0.1041	0.1345
NMF	0.7120	0.2926	**0.9949**	0.0843	0.1038	0.0754
BRPCA	0.9326	0.1624	0.9304	0.7847	0.0579	0.1079
SCMFDD	0.8988	0.0499	0.9915	0.162	0.0528	0.0856
LOMDA	0.8937	0.3252	0.3540	0.9431	0.0172	0.0338
RMDGCN	**0.9892**	0.8682	0.9836	0.7809	0.799	0.7897
THGC_MDA	0.9704	**0.9667**	0.9166	**0.9447**	**0.8951**	**0.9190**

The best results in each column are highlighted in bold.

### 3.3 Adjustment of parameters

**FIGURE 3:**
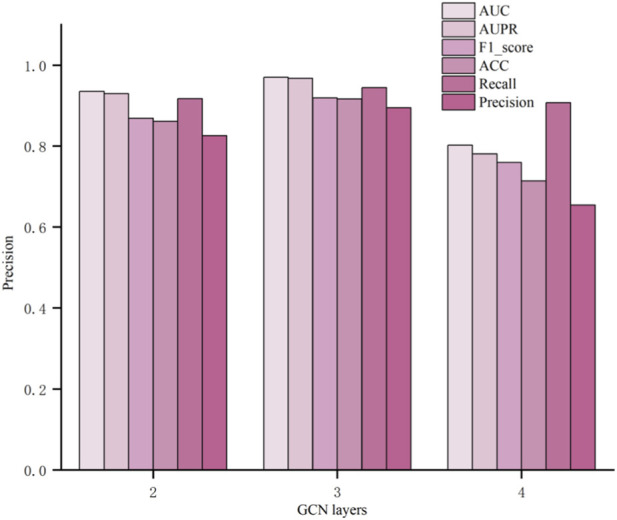
Comparison of adjusting the number of GCN layers.

If the number of GCN layers is too large, it will increase the model’s computational complexity, prolong training time, reduce the generalization ability, and raise the risk of overfitting (Kipf and Welling, 2017; Li et al., 2018). Increasing the number of GCN layers allows the model to aggregate information from higher-order neighbors. Conversely, if the number of GCN layers is too small, it may limit the model’s expressive ability and fail to capture more complex structures and patterns in the data, resulting in a decreased model performance. Therefore, it is necessary to investigate the impact of GCN layers count on the model to avoid problems caused by excessive or insufficient layers and achieve better performance. Experiments were conducted with 2, 3, and 4 GCN layers, and Figure 3 shows the evaluation metrics of the model with different GCN layer configurations. From the experimental results, it can be seen that when the number of GCN layers is 3, the model achieves the best predictive performance.

**FIGURE 4:**
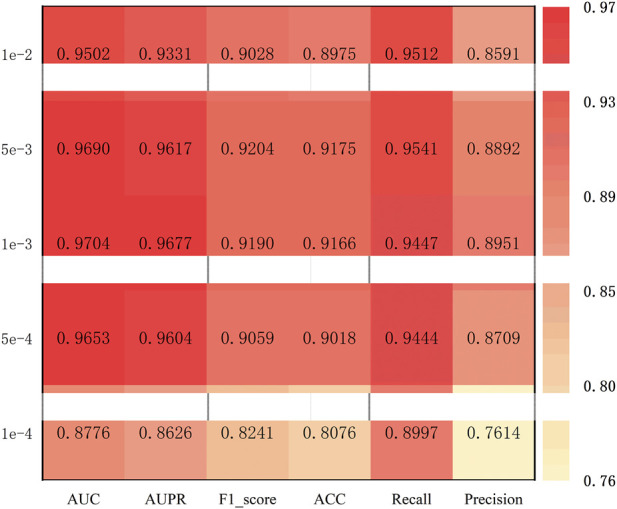
Performance comparison of adjusting learning rate.

The learning rate governs the step size for parameters updates during the training process. If the learning rate is too high or too low, it may significantly reduce the performance of the model. To identify the optimal value, the model was evaluated with learning rates of 1e-3, 5e-3, 1e-4, and 5e-4. As shown in Figure 4, the model achieves the best performance when the learning rate is 1e-3. Therefore, THGC_MDA sets the learning rate of 1e-3.

### 3.4 Ablation experiment

**FIGURE 5:**
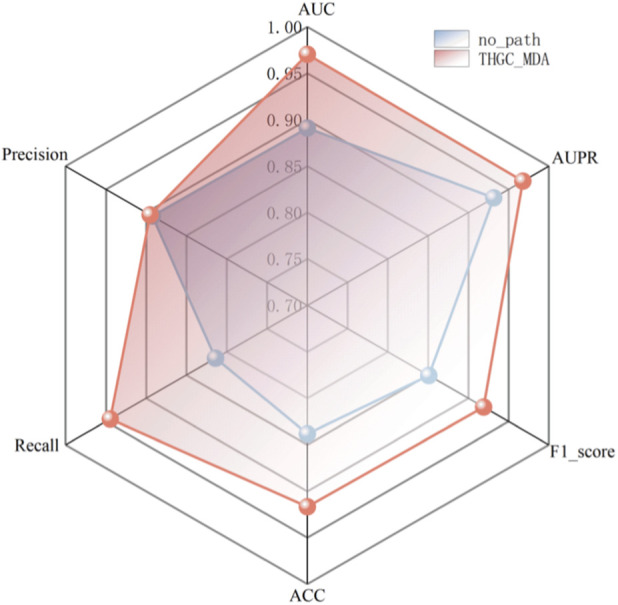
Comparison of ablation experiments.

To validate the effectiveness of constructing the three-node heterogeneous network in this paper, a meta-path ablation experiment was conducted. The adjacency matrix of m^1^A and diseases, as well as the similarity network of m^1^A and diseases, were directly predicted through GCN, without incorporating the new features fused via meta-paths. Experimental results show that the evaluation indicators showed an AUC of 0.8911 and an AUPR of 0.9316 in Figure 5, indicating that the meta-paths play a crucial role in the model’s results and can effectively improve the prediction performance of the model.

### 3.5 Case analysis

To further validate the model’s performance, we utilized THGC_MDA to predict potential m^1^A sites associated with renal cell carcinoma (RCC) and conducted Gene Ontology (GO) enrichment analysis on the host genes of these newly predicted sites. In this case study, all known RCC–m^1^A associations were masked as unknown, and the model was used to compute RCC–m^1^A prediction scores. Based on these scores, we ranked the RCC-related m^1^A sites and retrieved their corresponding host genes. After removing redundancies (several sites share the same host gene), 439 unique host genes remained with the criterion *p > 0.5*. These 439 genes were functionally annotated using GO terms from three dimensions: Cellular Component (CC), Biological Process (BP), and Molecular Function (MF).

**FIGURE 6:**
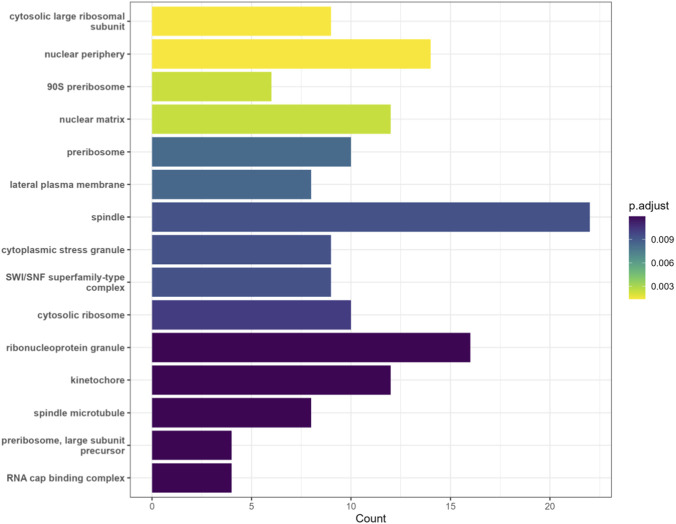
CC enrichment of genes related to RCC.

Figure 6 is the results of CC enrichment of genes related to RCC. Entries such as “lateral plasma membrane” represent key pathways for tumor migration and metastasis in RCC. Terms like “cytosolic large ribosomal subunit” and “preribosome” reflect aberrant translational machinery, which indicaes heightened proliferative activity of cancer cells, while “spindle” is associated with cell-cycle dysregulation and malignant proliferation. Clark et al. (Clark et al., 2019) demonstrated through multi-omic profiling that “cytosolic large ribosomal subunit” and “preribosome” are highly expressed in RCC, resulting in enhanced translational efficiency. Functional assays showed that inhibiting ribosome assembly attenuated cancer cell proliferation, confirming that aberrant ribosome translation drives enhanced proliferative activity in RCC.

**FIGURE 7:**
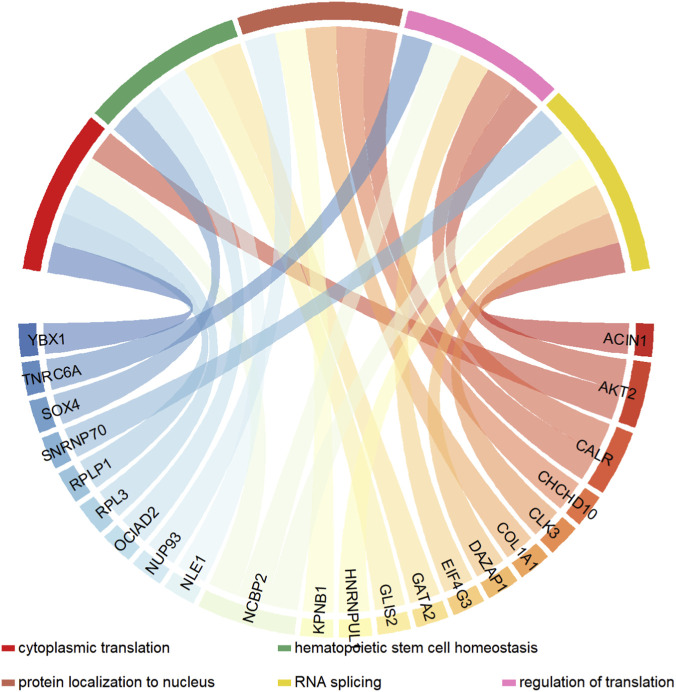
BP enrichment of genes related to RCC.

Many studies have demonstrated that the development of RCC is associated with various biological processes. We analyzed the enrichment relationship between BP and host genes, and the results are shown in Figure 7. From the figure, it can be seen that a single gene may be involved in multiple biological processes, while a biological process is driven by the interaction of multiple genes. In RCC, genes associated with RNA splicing and cytoplasmic translation are markedly activated, including core host genes such as RPL3 and YBX1. Previous studies have shown that piR-RCC (Wang et al., 2025) inhibits RCC proliferation and metastasis by blocking YBX1 nuclear translocation, thereby regulating its transcriptional repressor activity and indirectly affecting the expression of cytoplasmic translation-related genes.

**FIGURE 8:**
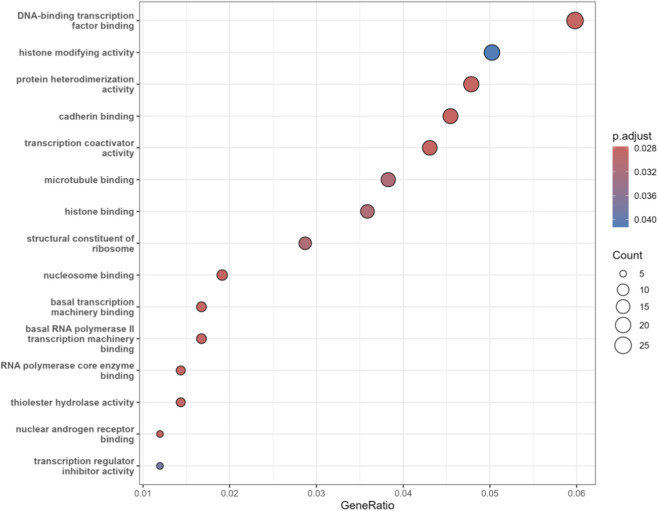
MF enrichment of genes related to renal cell carcinoma.

Next, we analyzed the MF category in the GO enrichment analysis. As shown in Figure 8, the larger the circle, the greater the number of genes enriched in this item. It can be observed that RCC is associated with histone modifying activity. This activity primarily refers to the chemical modification of histones, which alters chromatin compaction and regulates gene expression. In RCC, this mechanism is crucial for silencing tumor-suppressor genes (Zhu et al., 2019).

## 4 Conclusion

Identifying the association with m^1^A- related diseases can help us understand the crucial role of m^1^A modification in the pathogenesis of diseases. In this study, we propose a novel computational framework, THGC_MDA, to predict or discover the potential associations between m^1^A and diseases. Firstly, we obtain the disease similarity matrix by averaging the disease semantics and Jaccard similarity, and the m^1^A similarity matrix by averaging the m^1^A sequence similarity and Jaccard similarity matrix. To better identify the relationships between nodes, we introduced circRNA information and constructed the m^1^A meta-path network and disease meta-path network through the meta-path network. Then, we use GCN to extract deep embedding features of m^1^A and disease, and finally get the m^1^A-disease association prediction results through multilayer perceptron. In terms of predictive performance, the model achieved an AUC of 0.9684 and an AUPR of 0.9611 in the five-fold validation. Additionally, in the case of RCC, we verified the effectiveness of the model through GO enrichment analysis. However, since the model relies on circRNA as an intermediate bridge between m^1^A and the disease, and the annotation and functional research of circRNA are still insufficient at present, some missing data related to the association between certain diseases or m^1^A and circRNA may exist, thereby affecting the accuracy of the meta-path network construction. In the future, by integrating miRNA, lncRNA, proteins, etc., as intermediate bridges, multi-type meta-path networks (such as m^1^A→miRNA→disease, m^1^A→protein→disease, etc.) can be constructed. Through multi-path fusion algorithms, the comprehensive information mediated by multiple molecules is integrated, reducing the reliance on circRNA data.

Several limitations of this study should be acknowledged. First, although the proposed model demonstrates good predictive performance, it operates largely as a black-box model, which may limit its biological interpretability. Interpretability is crucial for understanding the underlying mechanisms of RNA modification–disease associations. In recent years, model interpretation methods such as SHAP (Lundberg and Lee, 2017) and lime (Ribeiro et al., 2016) have been widely used to explain machine learning models by estimating the contribution of input features to prediction outcomes. These approaches can quantify the influence of individual features and provide insights into the decision-making process of complex models. Second, all RNA modification-related variants in this study were obtained from the RMVar database (Luo et al., 2021), a comprehensive resource integrating experimentally validated and computationally predicted variants. However, relying on a single database may introduce potential biases, as data collection strategies, experimental conditions, and annotation methods may vary across studies.

To address these limitations, future work will pursue two complementary directions: integrating interpretability techniques into the proposed framework to analyze the contribution of different biological features, thereby identifying key factors influencing predicted m^1^A-disease associations and enhancing biological interpretability; and integrating data from multiple databases, such as RMDisease 2.0, as well as newly generated experimental datasets, to validate findings and improve the robustness and generalizability of the model. The combination of multi-database validation and model interpretability analysis will collectively strengthen the reliability of our predictions and provide deeper mechanistic insights into RNA-disease associations.

## Data Availability

The original contributions presented in the study are included in the article/supplementary material. The processed data and codes are freely available at GitHub https://github.com/XUEz-svg/THGC_MDA. Further inquiries can be directed to the corresponding authors.
